# Quantitative Analysis of the Effective Functional Structure in Yeast Glycolysis

**DOI:** 10.1371/journal.pone.0030162

**Published:** 2012-02-29

**Authors:** Ildefonso M. De la Fuente, Jesus M. Cortes

**Affiliations:** 1 Instituto de Parasitologia y Biomedicina Lopez-Neyra, CSIC, Granada, Spain; 2 Instituto de Investigaciones Sanitarias Biocruces, Barakaldo, Spain; 3 DECSAI: Departamento de Ciencias de la Computación e Inteligencia Artificial, Universidad de Granada, Granada, Spain; 4 CITIC: Centro de Investigación en Tecnologías de la Información y las Comunicaciones, Universidad de Granada, Granada, Spain; The Centre for Research and Technology, Hellas, Greece

## Abstract

The understanding of the effective functionality that governs the enzymatic self-organized processes in cellular conditions is a crucial topic in the post-genomic era. In recent studies, Transfer Entropy has been proposed as a rigorous, robust and self-consistent method for the causal quantification of the functional information flow among nonlinear processes. Here, in order to quantify the functional connectivity for the glycolytic enzymes in dissipative conditions we have analyzed different catalytic patterns using the technique of Transfer Entropy. The data were obtained by means of a yeast glycolytic model formed by three delay differential equations where the enzymatic rate equations of the irreversible stages have been explicitly considered. These enzymatic activity functions were previously modeled and tested experimentally by other different groups. The results show the emergence of a new kind of dynamical functional structure, characterized by changing connectivity flows and a metabolic invariant that constrains the activity of the irreversible enzymes. In addition to the classical topological structure characterized by the specific location of enzymes, substrates, products and feedback-regulatory metabolites, an effective functional structure emerges in the modeled glycolytic system, which is dynamical and characterized by notable variations of the functional interactions. The dynamical structure also exhibits a metabolic invariant which constrains the functional attributes of the enzymes. Finally, in accordance with the classical biochemical studies, our numerical analysis reveals in a quantitative manner that the enzyme phosphofructokinase is the key-core of the metabolic system, behaving for all conditions as the main source of the effective causal flows in yeast glycolysis.

## Introduction

Yeast glycolysis is one of the most studied dissipative pathways of the cell; it was the first metabolic system in which spontaneous oscillations were observed [1], [2], and the study of these rhythms allowed the construction of the first dynamic model where the kinetics of an enzyme was explicitly considered [3], [4].

Glycolysis is the central pathway of glucose degradation and it is implied in relevant metabolic processes, such as the maintenance of cellular redox states, the provision of ATP for membrane pumps and protein phosphorylation, biosynthesis, etc; and its activity is linked to a high variety of important cellular processes, e.g., glycolysis has a long history in cancer cell biology [5] and cell proliferation [6], there is a correlation between brain aerobic glycolysis and Amyloid-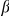 plaque deposition which might precede the clinical manifestations of the Alzheimer disease [7], the glycolytic inhibition abrogates epileptogenesis [8], and glycolysis is also related with oxidative stress [9] and apoptosis [10].

Over the last 30 years a large number of different studies focused on different molecular mechanisms allowing for the emergency of self-organized glycolytic patterns [11]–[15] and there is an international consensus about the functionality of glycolytic enzymes in yeast: the glycolytic system is a self-ordered metabolic structure, in which the functionally associated enzymes adopt a new supra-molecular configuration where some ordered metabolic dynamical patterns may arise. During the oxidation reaction from one molecule of glucose to two molecules of pyruvate, the glycolytic enzymes convert the potential biochemical energy to usable metabolic energy; a portion of this usable energy flows through the glycolytic system to maintain it far from the equilibrium. Beyond a critical point of instability of the non-equilibrium steady state it can emerge enzymatic activity rhythms. The sustained oscillations can be only maintained by the energy dissipation associated with the exchange of metabolites between the glycolytic system and its cellular environment. This type of non-equilibrium self-organization represents a dissipative structure and the metabolic oscillatory dynamics finds its roots in the non-linear regulatory processes which control the catalytic behavior of the glycolytic irreversible enzymes: the hexokinase, the phosphofructokinase and the pyruvatekinase [16], [17]. These functional structures which provide the temporal self-organization of metabolism correspond to dissipative systems, and the catalytic oscillatory behaviour finds its roots in the non-linear regulatory processes which control the dynamics of the irreversible enzymes [16], [17].

The theoretical basis of dissipative self-organization processes was formulated by Ilya Prigogine [18]. According to this theory, a dissipative structure is an open system which operates far from the thermodynamic equilibrium and it exchanges energy and matter with the external environment. As a consequence of these interchanging processes, spontaneous self-organization can emerge in the system producing high ordered spatial structures and temporal-functional metabolic patterns [17], [19].

From a dissipative point of view, the essential enzymatic states are those corresponding to the biochemical irreversible processes, and they are the only metabolic processes which might allow that the enzymatic system to work far from the thermodynamic equilibrium. Once the irreversible enzymatic system operates sufficiently far-from-equilibrium as the nonlinear nature of its kinetics, the steady state may become unstable leading to dynamical behaviours and new instabilities originating the emergence of different biochemical temporal patterns [20], [21].

In the yeast glycolysis, the main instability-generating mechanism is based on the self-catalytic regulation of the irreversible enzyme phosphofructokinase, specifically, the positive feed-back exerted by the reaction products, the ADP and fructose-1,6-bisphosphate [3], [16], [17], [22].

In this paper, to go a next step further in the understanding of the relationship between the classical topological structure and functionality we have analyzed the effective connectivity of yeast glycolysis, which in inter-enzyme interactions accounts for the influence that the activity of one enzyme has on the future of another [23]–[27].

For this purpose, we considered a yeast glycolytic model described by a system of three delay-differential equations in which there is an explicit consideration of the rate equations of the three irreversible enzymes hexokinase, phosphofructokinase and pyruvatekinase. These enzymatic activity functions were previously modeled and tested experimentally by other different groups [3], [28], [29].

We have obtained time series of enzymatic activity under different sources of the glucose input flux. The data corresponded to a typical quasi-periodic route to chaos which is in agreement with experimental conditions [30]. The dynamics of the glycolytic system changes substantially through this route, which allows for a better comparison of the enzymatic processes in periodic, quasi-periodic and chaotic conditions.

Using the non-linear analysis technique of Transfer Entropy [31], we have analyzed the glycolytic series and quantified the effective connectivity between enzymes. Transfer Entropy (TE) is based on methods to compute effective connectivity, which measures the causal influences between pairs of time series of enzymatic activity, thus resulting in an asymmetric quantity that defines a directionality in time from the cause to the effect. A number of measures have been proposed for the functionality and correlations between biochemical time series. However, functional correlations are symmetric measures and they do not imply effective connectivity as most synchronization measures do not distinguish between causal and non-causal interactions.

TE has been proposed as a rigorous, robust and self-consistent method for the effective connectivity i.e., causal quantification of the functional information flow among nonlinear processes [31]. Here we have applied the TE method to establish the effective functional connectivity of yeast glycolysis under dissipative conditions. The results show that in the numerical analysis of yeast glycolysis a effective functional structure emerges which is characterized by changing connectivity flows and a metabolic invariant that constraints the activity of the irreversible enzymes.

## Results

**Figure 1:**
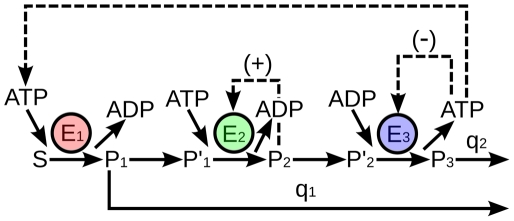
Multi-enzyme instability-generating system of yeast glycolysis. The main irreversible enzymatic processes are arranged in series: E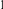 (hexokinase), E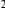 (phosphofructokinase) and E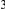 (pyruvatekinase). S, P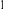, P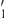, P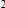, P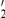 and P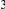 denote, respectively, the concentrations of glucose, glucose-6-phosphate, fructose 6-phospfate, fructose 1,6-bisphospfate, phosphoenolpyruvate and pyruvate. q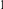 is the rate first-order constant for the removal of P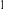; q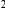 is the rate constant for the sink of the product P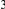. The model includes the feedback activation of E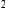 and the feedback inhibition of E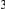. The ATP is consumed by E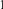 and recycled by E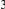.

In Fig. 1 it is represented the main enzymatic processes of yeast glycolysis (the irreversible stages) with the enzymes arranged in series.

The monitoring of the fluorescence of NADH in glycolyzing baker
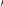
s yeast under sinusoidal glucose input flux, have shown that quasi-periodic time patterns are common at low amplitudes of the input and for high amplitudes chaotic behaviours emerge [32], [33].

In order to simulate these metabolic processes, the glycolytic system is considered under periodic input flux with a sinusoidal source of glucose 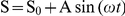. Assuming the experimental value of 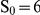 mM/h [34], after dividing by 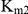 (the Michaelis constant of phosphofructokinase, see Methods for more details) we have obtained the normalized input flux 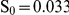 Hz.

**Figure 2:**
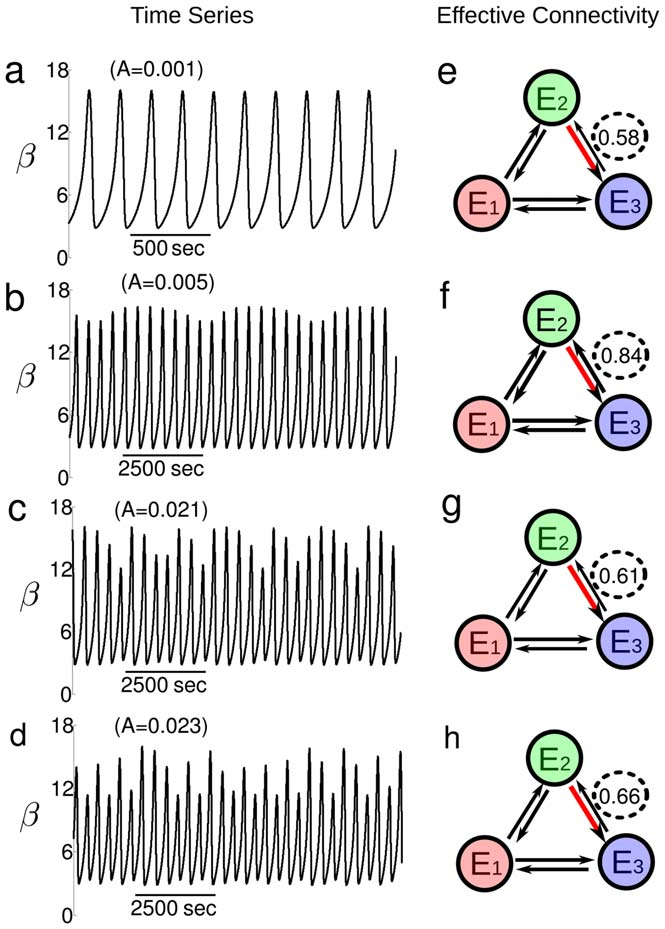
Glycolytic route to chaos and dynamical effective connectivity. a–d: The time evolution of the E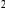 activity (the normalized concentration 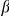, fructose 1,6-bisphospfate) shows a quasi-periodic route to chaos when varying the amplitude of the periodic input-flux from A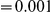 (top) to A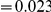 (bottom). (a) Periodic pattern. (b) Quasi-periodic oscillations. (c) Complex quasi-periodic motion indicating the beginning destruction of the periodic behavior. (d) Deterministic chaos. All series are plotted after 10000 seconds. e–h: Effective connectivity of the system for the same values of A in the left panel. The strength of effective connectivity is plotted with arrows width proportional to the Transfer Entropy divided by the maximum value (red arrow), results given in Table 1. Black dashed circles at the TE from E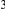 and E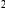 emphasize that the strength of Information flows is not the same, but varies through the quasi-periodic route to chaos.

Under these conditions, a wide range of different types of dynamic patterns can emerge as a function of the control parameter, hereafter the amplitude A of the sinusoidal glucose input flux [30], [35], [37]. In particular, it is observed a quasi-periodic route to chaos (cf. left panel in Fig. 2); thus for A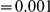 the biochemical oscillator exhibits a periodic pattern (Fig. 2a). An increment of the amplitude to A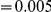 provokes a Hopf bifurcation generating another fundamental frequency, as a consequence, quasi-periodic behaviors emerge (Fig. 2b). Above A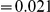, complex quasi-periodic oscillations appear (Fig. 2c). After a new Hopf bifurcation the originated dynamical behavior is not particularly stable and small perturbations produce deterministic chaos (A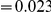, Fig. 2d), as predicted by Ruelle and Takens [38]. This route is in agreement with experimental conditions [30].

To go a next step further in the understanding of the relationship between the classical topological structure and effective functionality we have analyzed by means of non-linear statistical tools the catalytic patterns belonging to this scenario to chaos, and for each transition represented in the Fig. 2 we have obtained three time series corresponding to the variables 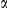, 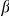 and 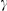 (12 in total), which denote respectively the normalized concentrations of glucose-6-phosphate, fructose 1-6-bisphosphate and pyruvate. Results are given in next sections.

### Effective functionality

Transfer Entropy (TE) quantifies the reduction in uncertainty that one variable has on its own future when adding another. This measure allows for a calculation of the functional influence in terms of effective connectivity between two variables [31]. The analysis of the glycolytic data by means of the TE method are shown in Table 1. The 4D vectors in square brackets correspond to the results obtained for the 4 different amplitudes of the considered glucose input flux, A = [0.001;0.005;0.021;0.023].

**Table 1:** Values of normalized Transfer Entropy.

	From E 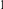	From E 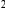	From E 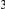
**To E** 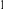	––	[0.73;0.88;0.72;0.76]	[0.74;0.80;0.68;0.74]
**To E** 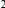	[0.76;0.80;0.70;0.72]	––	[0.58;0.84;0.61;0.66]
**To E** 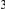	[0.78; 0.86;0.74;0.75]	[1.00;1.00;1.00;1.00]	––

The values of functional influence are ranging in 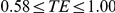, with mean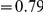 and standard deviation = 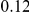, what indicates in general terms a high effective connectivity in the enzymatic system. The minimum value 0.58 corresponded to the causality flow between E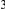 and E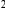 when a simple periodic behavior emerges. However, the functional connectivity from E2 to E3 shows the maximum value, achieved in all considered conditions of the glucose input flux.

The glycolytic effective connectivity is illustrated in Figs. 2e–h. The arrows width is proportional to the TE between pairs of enzymes. The values change through the quasi-periodic route to chaos, remarked from E3 to E2 by black dashed circles, [0.58;0.84;0.61;0.66].

In all cases analyzed, the values of TE present a maximum statistical significance (pvalue = 0, n = 50, Bonferroni Correction).

### Total Information flows and the functional invariant

**Figure 3:**
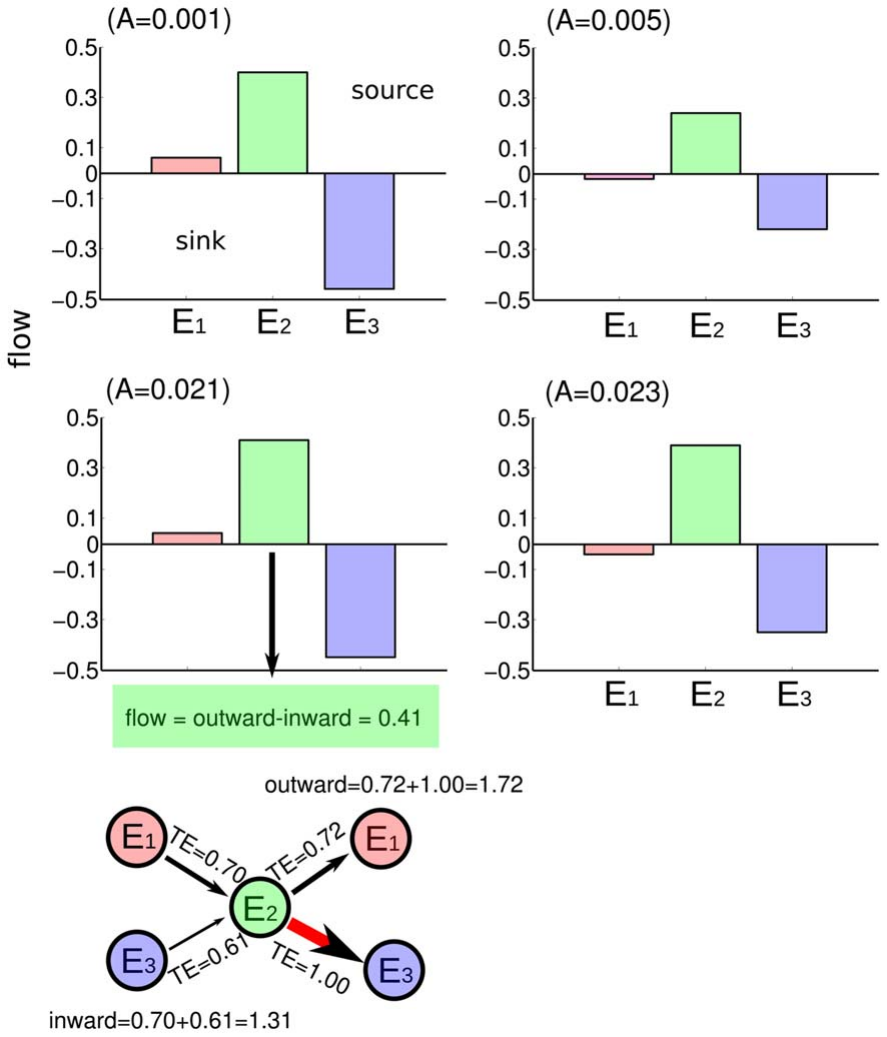
Total information flows and the functional invariant. Bars represent the total information flow, defined per each enzyme as the total outward TE minus the total inward. For A = 0.021 and E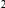 an schematic visualization of the calculation of this flow is shown (bottom graph of the panel). The functionality attributed for each enzyme is an invariant and preserved along the route, ie. E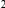 is a source, E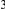 is a sink and E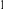 has a quasi-zero flow.

Next, we have measured the total information flow, defined as the total outward of Transfer Entropy arriving to one enzyme minus the total inward. Positive values mean that that enzyme is a source of causality flow and negative flows are interpreted as sinks or targets. The results of the total information flows are represented Fig. 3 and shown in Table 2. The maximum source of total transfer information (0.41) corresponds to the E2 enzyme (phosphofructokinase) for A = 0.021, when complex quasi-periodic oscillations appear in the glycolytic system.

**Table 2:** Values of total information flows.

**E** 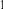	[0.06; −0.02;0.04;−0.04]	Quasi-zero flow
**E** 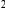	[0.40; 0.24;0.41;0.39]	source
**E** 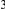	[−0.46; −0.22;−0.45;−0.35]	sink

For all conditions the enzyme E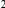 (phosphofructokinase) is the main source of effective influence and the enzyme E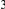 (pyruvatekinase) a sink, which could be interpreted as a target from the point of view of its effective functionality. The enzyme E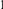 (hexokinase) is less constrained, and it has a flow close to zero for all conditions.

The attributed role to each enzyme, namely E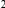 the source, E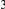 the sink and E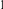 no-constrained is an invariant and preserved through the whole route to chaos.

### Functional Synchronization

Time correlations allows for quantification about how much two time series are statistically independent. According to that, we have measured the time pairwise correlations in the enzymatic system, and the corresponding results are shown in Table 3. The main finding is that E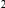 and E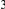 are highly synchronized (correlation = 0.90, pvalue = 0, Bonferroni Correction) and E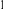 is anti-synchronized with both E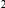 and E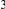 (respectively, correlation equals −0.65 and −0.66, pvalue = 0, Bonferroni Correction).

**Table 3:** Time Correlations.

	E 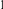	E 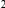	E 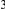
**E** 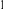	[1.00;1.00;1.00;1.00]	[−0.65;−0.66;−0.64;−0.63]	[−0.66;−0.66;−0.66;−0.66]
**E** 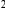	[−0.65;−0.66;−0.64;−0.63]	[1.00;1.00;1.00;1.00]	[0.90;0.90;0.90;0.90]
**E** 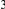	[−0.66;−0.66;−0.66;−0.66]	[0.90;0.90;0.90;0.90]	[1.00;1.00;1.00;1.00]

These values of time correlations were almost constant through the quasi-periodic route to chaos and established that the activities of E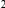 and E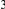 are grouped to the same function, being activated at similar time and oppositely to E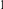.

### Redundancy and uncertainty reduction

The Mutual Information (MI) quantifies how much the knowledge of one variable reduces the entropy or uncertainty of the another [39]. The analysis of the glycolytic data by means of this method are shown in Table 4.

**Table 4:** Values of normalized Mutual Information.

	E 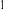	E 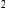	E 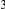
**E** 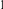	[1.00;1.00;1.00;1.00]	[0.52;0.49;0.45;0.44]	[0.48;0.49;0.45;0.44]
**E** 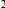	[0.52;0.49;0.45;0.44]	[0.85;0.84;0.85;0.86]	[0.47;0.46;0.45;0.45]
**E** 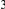	[0.48;0.49;0.45;0.44]	[0.47;0.46;0.45;0.45]	[0.76;0.74;0.76;0.78]

The high values of MI (close to 0.50) proved a high redundancy in information between the pairs of enzymes. In other words, the number of bits of information transferred from one enzyme to another is much larger than the actually needed.

The values in the principal diagonal of Table 4 represent the uncertainty for each variable. We have found these values gradually descending, H(E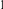) = [1.00;1.00;1.00;1.00], H(E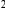) = [0.85;0.84;0.85;0.86] and H(E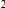) = [0.76;0.74;0.76;0.78], which is indicative of the uncertainty in the enzymatic activity patterns belonging to E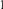, E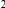 and E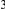 is reduced monotonously for all analyzed conditions.

The values of MI have a maximum statistical significance (pvalue = 0, n = 50, Bonferroni Correction).

Finally, we have computed the Mutual Information between the glucose input fluxes and the activity patterns of the different enzymes. In all cases, the MI was equal to zero, proving that the oscillations of the glucose were statistical independent of the glucose- 6-phosphate, fructose 1-6-biphosphate and pyruvate, products of the main irreversible enzymes of glycolysis.

## Discussion

In this paper we have quantified essential aspects of the effective functional connectivity among the main glycolytic enzymes in dissipative conditions.

First, we have computed under different source of glucose the causality flows in the metabolic system. This level of the functional influence accounts for the contribution of each enzyme to the generation of the different catalytic behavior and adds a directionality in the influence interactions between enzymes.

The results show that the flows of functional connectivity can change significantly during the different metabolic transitions analyzed, exhibiting high values of transfer entropy, and in all considered cases, the enzyme phosphofructokinase (E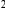) is the main source of effective causality flow; the pyruvatekinase (E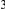) is the main sink of information flow; the hexokinase (E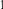) has a quasi-zero flow, meaning that, the total information arriving to E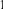 goes out to either E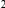 or E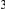.

The maximum source of total transfer information (0.41) corresponds to the E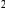 enzyme (phosphofructokinase) at the edge of chaos, when complex quasi-periodic oscillations emerge (cf. Fig. 2). This finding seems to be consistent with other studies which show that when a dynamical system operates in the frontier between order (periodic behavior) and chaos its complexity is maximal [40], [41].

The level of influence in terms of causal interactions between the enzymes is not always the same but varies depending on the substrate fluxes and the dynamic characteristics emerging in the system. In addition to the glycolytic topological structure characterized by the specific location of enzymes, substrates, products and regulatory metabolites there is an functional structure of information flows which is dynamic and exhibit notable variations of the causal interactions.

Another aspect of the glycolitic functionality was observed during the quantification of the Mutual Information, which measures how much the uncertainty about the one enzyme is reduced by knowing the other; we found that the uncertainty for E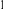, E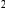 and E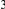 monotonously decreased for all the values of the periodic glucose input-flux.

Second, the numerical results show that for all analyzed cases the maximum effective connectivity corresponds to the Transfer Entropy from E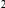 to E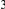, indicating the biggest information flow in the multi-enzyme instability-generating system. This is also corroborated by the measure of correlation between the different pairs of series which shows that E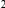 and E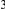 are highly correlated, or synchronized (correlation = 0.90 pvalue = 0) and E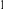 is anti-correlated with both E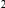 and E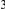 (respectively, correlation = −0.65 pvalue = 0 and correlation = −0.66 pvalue = 0). The values of time correlations establish that the activities of E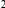 and E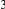 are grouped to the same function, being activated at similar time and oppositely to E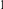.

Third, our analysis allows for a hierarchical classification in terms of what glycolytic enzyme is improving the future prediction of what others, and the results reveals in a quantitative manner that the enzyme E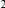 (phosphofructokinase) is the major source of causal information and represents the key-core of glycolysis. The second in importance is the E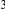 (pyruvatekinase).

From the biochemical point of view the E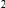 (phosphofructokinase) has been commonly considered as a major checkpoint in the control of glycolysis [42], [43]. The main reason for this generalized belief is that this enzyme exhibits a complex regulatory behavior that reflects its capacity to integrate many different signals [44]; from a dissipative point of view, this enzyme catalyzes a reaction very far from equilibrium and its self-catalytic regulation it has been considered the main instability-generating mechanism for the emergence of oscillatory patters in glycolysis [17]. The functional studies presented here give a quantification of the effective connectivity, and they confirm that the E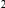 (phosphofructokinase) is the key-core of the pathway, and our results make stronger and expand the classical biochemical studies of glycolysis.

Forth, the dynamics of the glycolytic system changes substantially through the quasi-periodic route to chaos when the amplitude of the input-flux varies. However, the hierarchy obtained by transfer entropy, E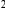 the flow, E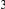 the sink and E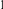 a quasi-zero flow, is preserved during this route and seems to be an invariant. This functional invariant of a metabolic process may be important for the understanding of functional enzymatic constraints in cellular conditions; but this issue requires other additional studies.

We want to emphasize that Transfer Entropy as a quantitative measure of effective causal connectivity can be a very useful tool in studies of enzymatic processes that operate far from equilibrium conditions. Moreover, many experimental observations have shown that the oscillations in the enzymatic activity seem to represent one of the most striking manifestations of the metabolic dynamic behaviors, of not only qualitative but also quantitative importance in cells (further details in Appendix S2).

Another interesting question is how our analysis can be scaled up from a single pathway to ensembles of pathways in which functional metabolic interactions between the catalytic sets can be derived. In concordance with this, we have constructed dissipative metabolic networks. Essentially, a Dissipative Metabolic Network (DMN) is an open system formed by a given set of dissipative enzymatic sets interconnected by substrate fluxes and three classes of regulatory signals: activatory (positive allosteric modulation), inhibitory (negative allosteric modulation) and all-or-nothing type (which correspond with the regulatory enzymes of covalent modulation). Certain enzymatic sets may receive an external substrate flux. In the DMN, the emergent output activity for each dissipative enzymatic set can be either oscillatory or steady state with an infinite number of distinct activity regimes. The first model of a Dissipative Metabolic Network was developed in 1999 [36], [45] which allowed to observe a singular spontaneously self-organized Systemic Metabolic Structure, characterized by a set of different enzymatic sets always locked into active states (metabolic core) while the rest of catalytic subsystems presented on-off dynamics. When an enzymatic subsystem is in an on-off state for a long time it can be turned on under specific metabolic conditions. In this first numerical work it was also suggested that the Systemic Metabolic Structure could be an intrinsic characteristic of metabolism, common to all living cellular organisms. Afterward, 2004 and 2005, several studies implementing flux balance analysis in experimental data produced new evidences of this Systemic Functional Structure [46], [47]. Specifically, it was observed a set of metabolic reactions belonging to different anabolic pathways which remain active under all investigated growth conditions. The rest of the reactions belonging to different pathways remain only intermittently active. These global catalytic processes were verified for Escherichia coli, Helicobacter pylori, and Saccharomyces cerevisiae [46], [47]. More recently, extensive analyses with different dissipative metabolic networks have shown that the fundamental factor for the spontaneous emergence of this systemic self-organized enzymatic structure is the number of enzymatic dissipative sets [48]. Moreover, it has been observed that the Systemic Metabolic Structure forms a unique dynamical system, in which self-organization, self-regulation and persistent properties may emerge [19]. In continuation with the glycolytic results presented here, we recently also applied TE analysis to these DMNs. We investigated the functional importance of the metabolic core belonging to a network of dissipative pathways and we found that the organization of the effective biomolecular information flows between the enzymatic sets is modular and the dynamical changes between the catalytic modules correspond to metabolic switches which allow critical transitions in the systemic enzymatic activity [49].

Transfer Entropy is able to detect the directed exchange of causality flows among the irreversible enzymes which might allow for a rigorous quantification of the effective functional connectivity of many dissipative metabolic processes in both normal and pathological cellular conditions.

The TE method applied to our numerical studies of yeast glycolisis shows the emergence of a new kind of dynamical functional structure which is characterized by changing connectivity flows and a metabolic invariant that constrains the activity of the irreversible enzymes.

## Methods

### Model

When the metabolite S (glucose) feeds the glycolytic system (Fig. 1), it is transformed by the first enzyme E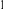 (hexokinase) into the product P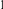 (glucose-6-phosphate). The enzymes E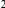 (phosphofructokinase) and E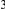 (pyruvatekinase) are allosteric, and transform the substrates P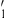 (fructose 6-phosphate) and P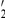 (phosphoenolpyruvate) in the products P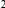 (fructose 1-6-bisphosphate) and P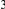 (pyruvate), respectively. The step P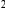 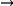
P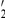 represents reversible activity processes, reflected in the dynamic system by the functional variable 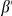. A part of P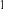 does not continue in the metabolic system, and is removed with a rate constant of q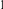 which is related with the activity of pentose phosphate pathway; likewise, q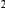 is the rate constant for the sink of the product P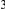 which is related with the activity of pyruvate dehydrogenase complex.

The main instability-generating mechanism in yeast glycolysis is the self-catalytic regulation of the enzyme E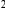 (phosphofructokinase), specifically, the positive feed-back exerted by the reaction products, the ADP and fructose-1,6-bisphosphate [3], [17], [22]. From a strictly biochemical point of view, E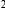 is also considered the main regulator enzyme of glycolysis [44]. The second irreversible stage for its regulatory importance is catalyzed by the enzyme E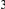 (pyruvatekinase) which is inhibited by the ATP reaction product [44]. Finally, the third irreversible process corresponds to the first stage the enzyme E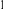 (hexokinase) which is dependent on the ATP.

In the determination of the enzymatic kinetics of the enzyme E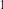 (hexokinase) the equation of the reaction speed dependent on glucose and ATP has been used [28]. The speed function of the allosteric enzyme E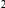 (phosphofructokinase) was developed in the framework of the concerted transition theory [3]. The reaction speed of the enzyme E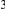 (pyruvatekinase), dependent on ATP and phospoenolpyruvate, was also constructed on the allosteric model of the concerted transition [29].

To study the kinetics of the dissipative glycolytic system we have considered normalized concentrations; 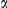, 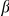 and 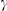 denoted respectively the normalized concentrations of P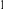, P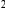 and P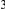. For a spatially homogeneous system the time-evolution is described by the following three delay differential equations:
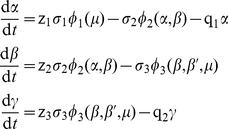
(1)where the functional variables 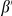 and 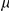 reflect the normalized concentrations of P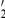 (phosphoenolpyruvate) and ATP respectively. The three main enzymatic functions are the following:
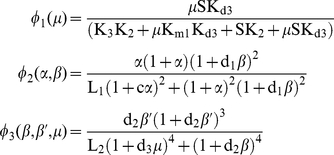
(2)and
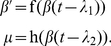
(3)The constants 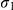, 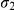 and 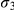 correspond to the maximum activity of E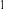, E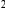 and E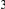 (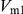, 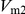 and 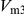) divided by the Michaelis constants of each enzyme, respectively 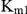, 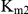 and 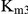. The constants z's are defined as 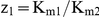, 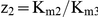 and 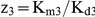, with 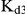 representing the dissociation constant of P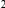 by E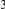. The constants d's are 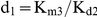, 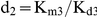 and 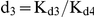, with 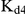 representing the dissociation constant of ATP; 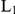 and 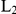 are respectively the allosteric constant of E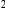 and E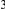; c is the non-exclusive binding coefficient of the substrate P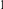. More details about parameter values and experimental references are given in Table S1.

From the dissipative point of view the essential enzymatic stages are those that correspond to the biochemical irreversible processes [21] and to simplify the model, we did not consider the intermediate part of glycolysis belonging to the enzymatic reversible stages. In this way, the functions f and h are supposed to be the identity function. Thus,
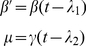
(4)


The initial functions present a simple harmonic oscillation in the following form:
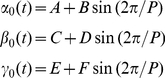
(5)with 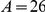, 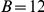, 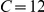, 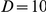, 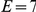, 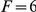 and 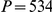.

The dependent variables 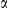, 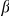 and 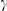 were normalized dividing them by 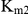, 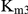 and 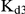, and the parameters 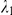 and 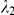 are time delays affecting the independent variable (further details in Appendix S1).

The numerical integration of the system was performed with the package ODE Workbench, which created by Dr. Aguirregabiria is part of the Physics Academic Software. Internally this package uses a Dormand-Prince method of order 5 to integrate differential equations. Further information at http://www.webassign.net/pas/ode/odewb.html.

This model has been exhaustively analyzed before, revealing a notable richness of emergent temporal structures which included the three main routes to chaos, as well as a multiplicity of stable coexisting states, see for more details [30], [35], [37].

### Transfer Entropy

TE allows for a quantification of how much the temporal evolution of the activity of one enzyme helps to improve the future prediction of another. The oscillatory patterns of the biochemical metabolites might have information which can be read-out by the TE. Further evidence about oscillatory behaviour in cellular conditions is given in Appendix S2.

For a convenient derivation, let generally assume that each of the pairs of enzymatic activity is represented by the two time series 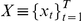 and 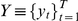. Here, 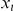 is the state value of the variable 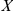 in time 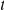, and similarly for 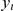. Let 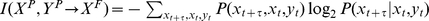 be the amount of information required to predict the future of 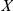 (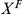) known both the pasts of 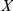 and 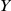 (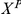 and 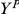). Analogously, let 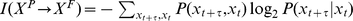 be the amount of information required to predict the future of 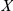 known only its past. The difference 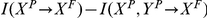 is by definition the transfer entropy from 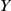 to 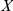, denoted by 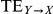. It quantifies the amount of information in digits that 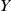 adds to the predictability of 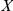.

Rewriting the conditional probabilities as the joint probability divided by its marginal, one obtains an explicit form for the Transfer Entropy:
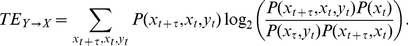
(6)


The problem of binning probabilities was solved by rounding at each time the values of the variables to the nearest integer, thus coarse-graining the continuous signal and counting the number of times (frequency) in which a variable is in a certain state. Similar choice were taking by Peng in his Matlab toolbox to compute the Mutual Information, further details in [50] and the web-site http://www.mathworks.com/matlabcentral/fileexchange/14888.

Another important parameter in the calculation of TE is the order 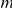 of the Markov process [31], which says about the number of past events one should consider in order to properly sample the stationary probabilities, ie. the future states in the time series depend on the past m states. We considered here different values of m, up to 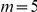 and results did not change considerably.

We also addressed the statistical significance for TE. This was achieved by comparing the obtained values of TE between two series of enzymatic activity, say X and Y, with the values obtained when considering a random permutation of the future of Y, what we called, the shuffled-future of Y. The values of TE in Table 1 were larger than those calculated in the shuffled situation (pvalue = 0, n = 50, Bonferroni Correction).

The formula (6) is fully equivalent to the Mutual Information between 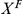 and 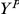 conditioned to 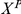. Thus, 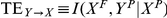, and consequently, Transfer Entropy says about how much information the inclusion of 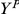 improves the prediction of 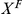 respect the situation of only considering 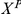, ie. 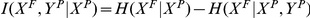. Therefore, TE is fully quantifying the information flows between pairs of variables. The values of TE were normalized between 0 and 1.

It is important to remark that the TE from 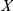 to 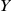 is different to the one from 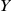 to 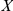, ie. the effective connectivity is asymmetric, adding a directionality in time which accounts for a particular case of *directed graphs*, the graph of information flows between pairs of enzymes.

Recently it was proved that the measures of effective connectivity based on Granger Causality [51] and Transfer Entropy coincide for Gaussian variables [52]. However, the glycolytic data in our model is not Gaussian (results not shown), thus the results of Transfer Entropy given in this paper might differ from those obtained by using an analysis based on Granger Causality.

### Mutual Information and Redundancy

MI quantifies how much the knowledge of one variable reduces the entropy or uncertainty of another. Therefore, MI says about how much information the two variables are sharing. Against other measures to compute correlations or statistical dependency, the strongest point of the MI is that it extends functionality to high order statistics [39]. Its definition is 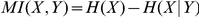, where 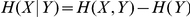 is the conditional entropy of 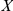 given 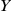. It accounts for the remaining uncertainty in 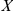 knowing the variable 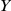. We referred 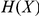 and 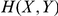 as respectively the joint and marginal (Shanon) entropies.

For statistical independent 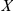 and 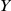 variables one has 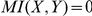. The other limit satisfies 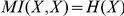, because of 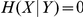. Therefore, the MI of two variables is bounded and satisfies that 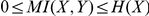. High values of MI mean that the redundancy in information between the two variables is large. The values of MI were normalized between 0 and 1.

## Supporting Information

Table S1 Model parameter values.
(PDF)

Appendix S1 Initial functions domains and phase shift.
(PDF)

Appendix S2 Metabolic oscillatory behavior in cellular conditions.
(PDF)
