# Supplementary material for: Quantitative Analysis of the Effective Functional Structure in Yeast Glycolysis
Source: PLoS One. 2012 Feb 29;7(2):e30162. doi: 10.1371/journal.pone.0030162 (PMC3290614; doi:10.1371/journal.pone.0030162)
Supplement: Table S1 — Model parameter values. (PDF) [file pone.0030162.s001.pdf]

Quantitative Analysis of the Effective Functional Structure in Yeast Glycolysis

Ildefonso M. De la Fuente and Jesus M. Cortes

Table S1: Model parameter values

Symbol	Value	Exp. References
V_{m1}	1.3×10^{-3} M/min	Ref. [1]
V_{m2}	1.2×10^{-2} M/min	Ref. [2]
V_{m3}	1.4×10^{-3} M/min	Ref. [3]
$\mathrm{K_{m1}}$	10.0×10^{-5}M	Ref. [4]
$\mathrm{K_{m2}}$	5.0×10^{-5}M	Ref. [5]
$\mathrm{K_{m3}}$	3.0×10^{-5}M	Ref. [6]
$\mathrm{K_2}$	6.3×10^{-5}M	Ref. [4]
$\mathrm{K_3}$	10.0×10^{-5}M	Ref. [4]
$\mathrm{K_{d2}}$	3.5×10^{-5}M	Ref. [7]
$\mathrm{K_{d3}}$	2.9×10^{-5}M	Ref. [8]
$\mathrm{K_{d4}}$	22.5×10^{-5}M	Ref. [9]
$\mathrm{L_1}$	6.0×10^{5}	Ref. [10]
$\mathrm{L_2}$	10^{3}	Ref. [9]
c	10^{-5}	Ref. [2]
$\mathrm{q_1}$	8.0×10^{-4}Hz	Free parameter
$\mathrm{q_2}$	6.9×10^{-2}Hz	Free parameter
ω	7.6×10^{-3}Hz	Free parameter
λ_1	2.0 sec	Free parameter
λ_2	0.6 sec	Free parameter

To the date the kinetics of individual enzymes in cellular conditions cannot be yet accurately determined. However, in-vitro studies give very precise quantifications of the enzymatic rate equations and their kinetic parameters. When in metabolic models it is considered the kinetics of irreversible enzymes, the kinetic laws observed in-vitro might work in-vivo as well. This does not mean that the in-vivo behavior is fully explained by in-vitro studies. But if one accounts for the dissipative conditions, as it can be done in numerical simulations, the in-vitro kinetic laws might be valid in physiological conditions. For these reasons, it is advisable to use dynamical models to study metabolism in which the molecular mechanisms of the irreversible enzymes are gathered by the in-vitro kinetics. These models take into account the dissipative mechanisms which are generating the instability present in far-from-equilibrium systems.

Therefore, an alternative to investigate the dynamics of metabolic pathways is to use the biochemical knowledge, gathered by the in-vitro kinetics of the irreversible enzymes, to predict the system catalytic behavior to eventually compare the prediction with experimental data [11].

References:

1. Gancedo J, Clifton D, Fraenkel D (1977) Yeast hexokinase mutants. J Biol Chem 252: 4443-4444.
2. Goldbeter A, Nicolis G (1976) An allosteric enzyme model with positive feedback applied to glycolytic oscillation. In Progress in Theoretical Biology. New York: Academic Press, pp. 65-160.
3. Markus M, Kuschmitz D, Hess B (1984) Chaotic dynamics in yeast glycolysis under periodic substrate input flux. FEBS Lett 172: 235-238.
4. Viola ER, Raushel MF, Rendina RA, Cleland WW (1982) Substrate synergism and the kinetic mechanism of yeast hexokinase. Biochemistry 21: 1295- 1302.
5. Bartrons R, Schaftingen E, Vissers S, Hers H (1982) The stimulation of yeast phosphofructokinase by fructose 2,6bisphosfate. FEBS Lett 143: 137-140.
6. Waygood BE, Sanwal DB (1974) The control of pyruvatekinase of Escherichia coli. J Biol Chem 249: 265-274.
7. Laurent M, Dessen P, Seydoux JF (1979) Allosteric regulation of yeast phosphofructokinase. J Biol Chem 254: 7515-7520.
8. Markus M, Plesser T, Boiteux A, Hess B, Malcovati M (1980) Rate law of pyruvate kinase type. 1 from Escherichia coli. Biochem J 189: 421-433.
9. Boiteux A, Markus M, Plesser T, Hess B (1983) Interaction of pyruvate kinase from Escherichia coli with fructose l,6-bisphosphate and calcium ions. Biochem J 211: 631-640.
10. Blangy D, But H, Monod J (1968) Kinetics of the ahosteric interactions of phosphofructokinase from Escherichia coli. J Mol Biol 31: 13-35.

11. Teusink B, Passarge J, Reijenga CA, Esgalhado E, van der Weijden CC, et al. (2000) Can yeast glycolysis be understood in terms of in vitro kinetics of the constituent enzymes? Testing biochemistry. Eur J Biochem 267: 5313-5329.
