# Supplementary material for: Quantitative Analysis of the Effective Functional Structure in Yeast Glycolysis
Source: PLoS One. 2012 Feb 29;7(2):e30162. doi: 10.1371/journal.pone.0030162 (PMC3290614; doi:10.1371/journal.pone.0030162)
Supplement: Appendix S1 — Initial functions domains and phase shift. (PDF) [file pone.0030162.s002.pdf]

Quantitative Analysis of the Effective Functional Structure in Yeast Glycolysis

Ildefonso M. De la Fuente and Jesus M. Cortes

Appendix S1: Initial functions domains and phase shift

The modeling based on ordinary differential equations (ODE) is a common feature in most of the non-linear dynamic studies of metabolic systems. According to that, self-organized dynamic behavior is considered to depend on the different values achieved by the parameters linked to the dependent variables. Moreover, the initial conditions are always constant values (never initial functions) and when determining the particular solutions, only a small number of degrees of freedom are available. Consistently with the theory of dynamical systems, delay processes can be approximated accurately by augmenting the original variables with other auxiliary functional variables. By means of these systems of functional differential equations with delay it is possible to consider initial functions (instead of the constant initial values of ODE systems) and to analyze the consequences that the variations in the parametric values linked to the independent variable (time) have upon the integral solutions of the system. A typical ODE system is written as:

$$\begin{aligned}
y_1'(t) &= f_1(y_1(t), y_2(t), \ldots, y_n(t)) \\
y_2'(t) &= f_2(y_1(t), y_2(t), \ldots, y_n(t)) \\
&\vdots \\
y_n'(t) &= f_n(y_1(t), y_2(t), \ldots, y_n(t))
\end{aligned} \tag{1}$$

and a dynamic model governed by a delayed functional differential equations system can be expressed as:

$$\begin{aligned} y_1'(t) &= f_1(z_1(t), \ldots, z_r(t), y_{r+1}(t), y_{r+2}(t), \ldots, y_n(t)) \\ y_2'(t) &= f_2(z_1(t), \ldots, z_r(t), y_{r+1}(t), y_{r+2}(t), \ldots, y_n(t)) \\ &\vdots \\ y_n'(t) &= f_n(z_1(t), \ldots, z_r(t), y_{r+1}(t), y_{r+2}(t), \ldots, y_n(t)) \end{aligned} \tag{2}$$

where the dependent variable is a n-dimensional vector of the form $y = (y_1, y_2, \ldots, y_n$, and the delayed variables are represented by $z_i = h_i(t - \lambda_i)$, λ_i are the time delays and h_i arbitrary functions. In system of Eqs. (2) the first derivatives of $y_1, y_2, \ldots, y_n$ are related to the variables $y_1, y_2, \ldots, y_r$ evaluated in $t - \lambda_r$ and related to $y_{r+1}, y_{r+2} \ldots, y_n$ evaluated in t.

Therefore in delayed differential equations, unlike ODE systems, to determine a particular solution in the interval $[t_0 - \delta, t_0]$ with $\delta = \max(\lambda_1, \ldots, \lambda_r)$ it is necessary to give the value of the solution f_0 in such interval. That involves the evaluation of the functions $f_0 : [t_0 - \delta, t_0] \rightarrow \Re^n$, called initial functions. It can be observed therefore that infinite degrees of freedom exist in the determination of the particular solutions. In the system described by (2) it is possible to take the initial function f_0 equal to any $y(t)$, which, in particular, can be a periodic solution of the system (2) for $\lambda_1 = \lambda_2 = \cdots = \lambda_r = 0$ and $t \leq t_0$. The initial function will be $y^\delta(t) : [t_0 - \delta, t_0] \rightarrow \Re^n$, with $y^\delta(t) = y(t)\ \forall t \in [t_0 - \delta, t_0]$.

In the particular case of $\lambda_1 > 0$ and $\lambda_2 = \cdots = \lambda_r = 0$ the first component of the initial function satisfies that $y_1^\delta(t) : [t_0 - \delta, t_0] \rightarrow \Re$; each λ_1 corresponds to $y_1^\delta(t_0 - \lambda_1)$, the first component of y in time $t_0 - \lambda_1$. The parameter λ_1 determines the initial function domain, and given the solution is periodic, for each different domain of the initial function exists an ordinate value in the origin $y_1^\delta(t_0 - \lambda_1)$ and a corresponding phase-shift. It is observed that $y_1^\delta(t_0 - \lambda_1)$ is the value of the function $y_1^\delta(t - \lambda_1)$ evaluated in t_0 where the function exhibits an initial function with a phase shift of λ_1.

With this type of systems, it is possible to account the dynamical behavior related to parametric variations linked to the independent variable. The parametric variations λ_i affect the independent variable which represent time delays and can be related to the phase shifts and the domains of the initial functions [1,2,3].

References:

1. De la Fuente IM, Martinez L, Veguillas J, Aguirregabiria J (1996) Quasiperiodicity route to chaos in a biochemical system. Biophys J 71: 2375-2379.

2. De la Fuente IM, Martinez L, Veguillas J (1996) Intermittency route to chaos in a biochemical system. Biosystems 39: 87-92.

3. De la Fuente IM (1999) Diversity of temporal self-organized behaviors in a biochemical system. BioSystems 50: 83-97.
