# Supplementary material for: Quantitative Analysis of the Effective Functional Structure in Yeast Glycolysis
Source: PLoS One. 2012 Feb 29;7(2):e30162. doi: 10.1371/journal.pone.0030162 (PMC3290614; doi:10.1371/journal.pone.0030162)
Supplement: Appendix S2 — Metabolic oscillatory behavior in cellular conditions. (PDF) [file pone.0030162.s003.pdf]

# Quantitative Analysis of the Effective Functional Structure in Yeast Glycolysis

Ildefonso M. De la Fuente and Jesus M. Cortes

## Appendix S2: Metabolic oscillatory behavior in cellular conditions

During the last four decades, extensive studies of dynamical biochemical behaviors in cellular conditions both in prokaryotic and eukaryotic cells have shown the spontaneous emergence of molecular oscillations in most of the fundamental metabolic processes. For instance, there are oscillatory biochemical processes involved in: NAD(P)H concentration [1], biosynthesis of phospholipids [2], cyclic AMP concentration [3], ATP [4] and other adenine nucleotide levels [5], intracellular glutathione concentration [6], actin polymerization [7], ERK/MAPK metabolism [8], mRNA levels [5], intracellular free amino acid pools [9], cytokinins [10], cyclins [11], transcription of cyclins [12], gene expression [13-16], microtubule polymerization [17], membrane receptor activities [18], membrane potential [19], intracellular pH [20], respiratory metabolism [21], glycolysis [22], intracellular calcium concentration [23], metabolism of carbohydrates [24], beta-oxidation of fatty acids [25], metabolism of mRNA [26], tRNA [27], proteolysis [28], urea cycle [29], Krebs cycle [30], mitochondrial metabolic processes [31], nuclear translocation of the transcription factor [32], amino acid transports [33], peroxidase-oxidase reactions [34], protein kinase activities [35] and photosynthetic reactions [36].

The transition from simple periodic behavior to complex oscillatory phenomena, including bursting (oscillations with one large spike and series of secondary oscillations) [37] and chaos (irregular oscillations)[38] is often observed in metabolic behaviors.

In the conditions prevailing inside the cell, the oscillations seem to represent one of the most striking manifestations of dynamic behavior, of not only qualitative but also quantitative importance in cell metabolic systems; e.g., considering only the transcription processes, it has been reported that at least 60% of all gene expression in Saccharomyces cerevisae oscillate with an approximate period of 300 min [39] and at least 10% of the rest of cellular transcripts oscillate in a circadian manner [40].

This new type of supra-molecular self-organization that operates in far from equilibrium conditions was called dissipative structures by Prigogine [41,42] and the enzymatic functional structures that provide the temporal self-organization of metabolism find their roots in the many regulatory processes that control the dynamics of the enzymes that belong to them [42,43].

The dissipative structure constitutes a fundamental element to understand the emergence of the spatial-functional architecture in cells and provide a conceptual framework that allows us to unify the dynamic, self-organized metabolic processes that occur in all biological organisms [44]. On the other hand, it was also suggested using dissipative metabolic networks that a dynamic global metabolic structure could be present in all living cells [45-49].

Quantitative studies by means of non-linear tools and computational approaches are particularly valuable for exploring dynamic phenomena associated with dissipative metabolic structures, and these methods will be crucial in making sense of the functional metabolic architecture of the cell [44].

## References:

1. Rosenspire AJ, Kindzelskii AL, H. Petty (2001) Pulsed DC electric fields couple to natural NAD(P)H oscillations in HT-1080 fibrosarcoma cells. J Cell Sci 114: 1515-1520.

2. Marquez S, Crespo C, Carlini V, Garbarino-Pico E, Baler R et al. (2001) The metabolism of phospholipids oscillates rhythmically in cultures of fibroblasts and is regulated by the clock protein PERIOD 1. FASEB J 18: 519-521.

3. Holz GG, Heart EE, Leech CA (2008) Synchronizing $Ca^{2+}$ and cAMP oscillations in pancreatic beta cells: a role for glucose metabolism and GLP-1 receptors? Am J Physiol Cell Physiol 294:c4-c6.

4. Ainscow EK, Mirsham S, Tang T, Ashford MLJ, Rutter GA (2002) Dynamic imaging of free cytosolic ATP concentration during fuel sensing by rat hypothalamic neurones: evidence for ATPindependent control of ATP-sensitive $K^{+}$ channels. J Physiol 544: 429-445.

5. Xu Z, Yaguchi S, Tsurugi K (2004) Gts1p stabilizes oscillations in energy metabolism by activating the transcription of TPS1 encoding trehalose-6-phosphate synthase 1 in the yeast Saccharomyces cerevisiae. Biochem J 383: 171178.

6. Lloyd D, Murray DB (2005) Ultradian metronome: timekeeper for orchestration of cellular coherence. TRENDS in Biochem Sci 30: 373-377.

7. Rengan R, Omann GM (1999) Regulation of Oscillations in Filamentous Actin Content in Polymorphonuclear Leukocytes Stimulated with Leukotriene B4 and Platelet-Activating Factor. Biochem and Biophys Res Comm 262: 479486.

8. Shankaran H, Ippolito DL, Chrisler WB, Resat H, Bollinger K et al. (2009) Rapid and sustained nuclearcytoplasmic ERK oscillations induced by epidermal growth factor. Mol Sys Biol Article 5: 332.

9. Hans MA, Heinzle E, Wittmann CH (2003) Free intracellular amino acid pools during autonomous oscillations in Saccharomyces cerevisiae. Biotechnol Bioeng 82: 143-151.

10. Hartig K, Beck E (2005) Endogenous Cytokinin Oscillations Control Cell Cycle Progression of Tobacco BY-2 Cells Plant Biology 7: 33-40.

11. Hungerbuehler AK, Philippsen P, Gladfelter AS (2007) Limited Functional Redundancy and Oscillation of Cyclins in Multinucleated Ashbya gossypii Fungal Cells Eukaryot Cell 6: 473486.

12. Shaul O, Mironov V, Burssens S, Van Montagu M, Inze D (1996) Two Arabidopsis cyclin promoters mediate distinctive transcriptional oscillation in synchronized tobacco BY-2 cells. Proc Natl Acad Sci USA 93: 48684872.

13. Chabot JR, Pedraza JM, Luitel P, van Oudenaarden A (2007) A Stochastic gene expression out-of-steady-state in the cyanobacterial circadian clock. Nature 450: 1249-1252.

14. Tian B, Nowak DE, Brasier AR (2005) A TNF-induced gene expression program under oscillatory NF-B control. BMC Genomics 6: 137.

15. Tonozuka H, Wang J, Mitsui K, Saito T, Hamada Y et al. (2001) Analysis of the Upstream Regulatory Region of the GTS1 Gene Required for Its Oscillatory Expression. J Biochem 130: 589-595.

16. Klevecz RR, Bolen J, Forrest G, Murray DB (2004) A genomewide oscillation in transcription gates DNA replication and cell cycle. Proc Natl Acad Sci USA 101: 1200-1205.

17. Lange G, Mandelkow EM, Jagla A, Mandelklow E (2004) Tubulin oligomers and microtubule oscillations Antagonistic role of microtubule stabilizers and destabilizers. FEBS 178: 61-69.

18. Placantonakis DG, Welsh JP (2001) Two distinct oscillatory states determined by the NMDA receptor in rat inferior olive. J Physiol 534: 123-140.

19. De Forest M, Wheeler CJ (1999) Coherent Oscillations in Membrane Potential Synchronize Impulse Bursts in Central Olfactory Neurons of the Crayfish. J Neurophysiol 81: 1231-1241.

20. Sanchez-Armass S, Sennoune SR, Maiti D, Ortega F, Martinez-Zaguila R (2006) Spectral imaging microscopy demonstrates cytoplasmic pH oscillations in glial cells. Am J Physiol Cell Physiol 290: C524-C538.

21. Lloyd D, Eshantha L, Salgado J, Turner MP, Murray DB (2002) Respiratory oscillations in yeast: clock-driven mitochondrial cycles of energization. FEBS Lett 519: 41-44.

22. Danu S, Surensen PG, Hynne F (1999) Sustained oscillations in living cells. Nature 402: 320-322.

23. Ishii K, Hirose K, Iino M (2006) $Ca^{2+}$ shuttling between endoplasmic reticulum and mitochondria underlying $Ca^{2+}$ oscillations. EMBO 7: 390-396.

24. Jules M, Francois J, Parrou JL (2005) Autonomous oscillations in Saccharomyces cerevisiae during batch cultures on trehalose. FEBS J 272: 1490-1500.

25. Getty L, Panteleon AE, Mittelman SD, Dea MK, Bergman RN (2000) Rapid oscillations in omental lipolysis are independent of changing insulin levels in vivo. J Clin Invest 106: 421-430.

26. Klevecz RR, Murray DB (2001) Genome wide oscillations in expression Wavelet analysis of time series data from yeast expression arrays uncovers the dynamic architecture of phenotype. Mol Biol Rep 28: 73-82.

27. Brodsky V, Boikov PY, Nechaeva NV, Yurovitsky YG, Novikova TE et al. (1992). The rhythm of protein synthesis does not depend on oscillations of ATP level. J Cell Sci 103: 363-370.

28. Kindzelskii AL, Zhou MJ, Haugland PR, Boxer AL, Petty RH (1998) Oscillatory Pericellular Proteolysis and Oxidant Deposition During Neutrophil Locomotion. Biophys J 74: 9097.

29. Fuentes JM, Pascual MR, Salido G, Soler G, Madrid JA (1994) Oscillations in rat liver cytosolic enzyme activities of the urea cycle. Archiv Physiol And Biochem 102: 237-241.

30. Wittmann C, Hans M, Van Winden AW, Ras C, Heijnen JJ (2005) Dynamics of intracellular metabolites of glycolysis and TCA cycle during cell-cycle-related oscillation in Saccharomyces cerevisiae. Biotechnol Bioeng 89: 839-847.

31. Aon MA, Roussel MR, Cortassa A, ORourke B, Murray DB et al. (2008) The scale-free dynamics of eukaryotic cells. Plos One 3: e3624.

32. Garmendia-Torres C, Goldbeter A, Jacquet M (2007) Nucleocytoplasmic Oscillations of the Yeast Transcription Factor Msn2: Evidence for Periodic PKA Activation. Current Biology 17: 1044-1049.

33. Barril EF, Potter AR (1968) Systematic Oscillations of Amino Acid Transport in Liver from Rats Adapted to Controlled Feeding Schedules. J Nutrition 95: 228-237.

34. Moller AC, Hauser MJB, Olsen LF (1998) Oscillations in peroxidase-catalyzed reactions and their potential function in vivo. Biophys Chem 72: 63-72.

35. Chiam HK, Rajagopal G (2007) Oscillations in intracellular signaling cascades. Phys Rev E 75: 061901.

36. Smrcinova M, Sorensen PG, Krempasky J, Ballo P (1998) Chaotic oscillations in a chloroplast system under constant illumination. Int J Bifur Chaos 8: 2467-2470.

37. Dekhuijzen A, Bagust J (1996) Analysis of neural bursting: Nonrhythmic and rhythmic activity in isolated spinal cord. J Neurosci Methods 67: 141147.

38. Olsen LF, Degn H (1985) Chaos in biological systems. Q Rev Biophys 18: 165225.

39. Tu BP, Kudlicki A, Rowicka M, McKnight SL (2005) Logic of the yeast metabolic cycle: Temporal compartmentalization of cellular processes. Science 310: 11521158.

40. Nakahata Y, Grimaldi B, Sahar S, Hirayama J, Sassone-Corsi P (2007) Signaling to the circadian clock: Plasticity by chromatin remodelling. Curr Opin Cell Biol 19: 230237.

41. Nicolis N, Prigogine I (1977) Self-organization in nonequilibrium systems. From dissipative structures to order through fluctuations. New York: J. Wiley & Sons.

42. Goldbeter A (2007) Biological rhythms as temporal dissipative structures. Adv Chem Phy 135: 253-295.

43. Goldbeter A (2002) Computational approaches to cellular rhythms. Nature 420: 238245.

44. De la Fuente IM (2010) Quantitative Analysis of Cellular Metabolic Dissipative, Self-Organized Structures. Int J Mol Sci 11: 3540-3599.

45. Almaas E, Kovacs B, Vicsek T, Oltvai ZN, Barabasi AL (2004) Global organization of metabolic fluxes in the bacterium Escherichia coli. Nature 427: 839-843.

46. Almaas E, Oltvai ZN, Barabasi AL (2005) The activity reaction core and plasticity of metabolic networks. PLoS Comput Biol 1:e68.

47. De la Fuente IM, Martinez L, Perez-Samartin AL, Ormaetxea L, Amezaga C et al. (2008) Global Self-organization of the cellular metabolic structure. Plos One 3: e3100.

48. De la Fuente IM, Vadillo F, Perez-Pinilla MB, Vera-Lopez A, Veguillas J (2009) The number of catalytic elements is crucial for the emergence of metabolic cores. Plos One 4: e7510.

49. De la Fuente IM, Vadillo F, Perez-Samartin AL, Perez-Pinilla MB, Bidaurrazaga J et al. (2010) Global self-regulations of the cellular metabolic structure. Plos One 5: e9484.
